# Effect of physical exercise on the emotional and cognitive levels of patients with substance use disorder: a meta-analysis

**DOI:** 10.3389/fpsyg.2024.1348224

**Published:** 2024-02-09

**Authors:** Yamiao Zheng, Yiyang Zhao, Xilian Chen, Shanshan Li

**Affiliations:** ^1^School of Sports Science, Qufu Normal University, Qufu, China; ^2^School of Physical Education, Sichuan University, Chengdu, China

**Keywords:** physical exercise, substance use disorder, emotion, cognition, meta-analysis

## Abstract

**Introduction:**

This study investigated the impact of different modes of physical exercise on the emotional and cognitive levels of patients with Substance Use Disorder (SUD). By exploring the most effective intervention types, cycle, frequency, and duration, we aimed to provide evidence-based recommendations for the adjunctive treatment of SUD.

**Methods:**

We conducted a systematic search in five databases, including PubMed, Web of Science, The Cochrane Library, ScienceDirect, and EBSCO, from database inception up to May 2023, and identified 4,255 randomized controlled trials addressing the influence of physical exercise on the emotional and cognitive levels of SUD patients. Data extraction and analysis were performed using Review Manager 5.4 software, focusing on 11 studies that met the inclusion criteria and included 895 participants. Subsequently, a meta-analysis was conducted using Stata 16.0 software, presenting the results in the form of standardized mean differences (SMD) and 95% confidence intervals (CI).

**Results:**

Our findings indicate that physical exercise significantly alleviates anxiety and depression in SUD patients while improving their cognitive function. Specifically, physical exercise was found to reduce anxiety (SMD = −0.726 [−1.349, −0.103], *p* < 0.05) and depression (SMD = −0.666 [−1.077, −0.255], *p* < 0.05) and enhance cognitive levels (SMD = −0.523 [−0.887, −0.159], *p* < 0.05) among patients. Subgroup analysis further revealed that SUD patients benefitted most from physical exercise when engaging in aerobic exercises lasting over 12 weeks, with a frequency exceeding 40 sessions and each session lasting more than 60 min.

**Discussion:**

In conclusion, our study affirms that physical exercise mitigates anxiety and depression while enhancing cognitive function in SUD patients, making it an effective measure for adjunctive clinical treatment.

## 1 Introduction

The Diagnostic and Statistical Manual of Mental Disorders 5th edition (American Psychiatric Association, 2013) combines the DSM-IV categories of “substance dependence” and “substance abuse” into a single entity called “Substance Use Disorder” (SUD) (American Psychiatric Association, 2013), which is also known as drug addiction. According to the World Drug Report (United Nations Office on Drugs and Crime, 2022), there were over 296 million drug users worldwide in 2021, of which approximately 13.2 million injected drugs. The number of individuals with SUD has surged to 39.5 million, representing a 45% increase over the previous decade. SUD activates the brain’s reward system, inducing pleasure, whose specific characteristics vary by substance, such as alcohol, caffeine, cannabis (synthetic cannabinoids), hallucinogens (e.g., methamphetamine), inhalants, opioids, sedatives, hypnotics, anxiolytics, central nervous system stimulants (e.g., amphetamines, caffeine), and tobacco (Sherer et al., 2023). SUD depletes dopamine stores in the brain, impairs dopamine and serotonin terminal binding, and leads to withdrawal symptoms; this causes anxiety, depression, and cognitive decline and creates severe negative emotional states (Brellenthin and Koltyn, 2016). SUD involves adaptive changes in brain neurons to counteract the acute reinforcing effects of drugs (Self and Nestler, 1995), thus posing severe health problems and exerting a negative impact on the lives of individuals (Kohlerova et al., 2023). Existing treatments for SUD primarily rely on medication, focusing on receptor agonists (e.g., nicotine replacement therapy for tobacco use disorder, methadone and buprenorphine for opioid use disorder); however, overuse can lead to medication addiction (NIH, 2020).

Physical exercise has shown significant efficacy as an adjunct intervention for SUD patients (Dowla et al., 2022). Specifically, short-term aerobic exercise has been found to improve drug cravings and reduce inhibitory deficits in methamphetamine users (Wang et al., 2019). Long-term physical exercise interventions also play a positive role in assisting the treatment of substance addiction (Wang et al., 2022); however, physical exercise must be performed regularly and continuously to observe the long-term physiological and psychological changes in the bodies of SUD patients (Williams et al., 2011). While theoretical and empirical research indicate that both short- and long-term aerobic exercise can significantly reduce drug cravings, lower the desire for substance use, improve inhibitory control (Lynch et al., 2013; Wang et al., 2014), and enhance the circulation of amines in SUD individuals, the optimal intervention duration for adjunctive treatment of SUD patients has not yet been established. Moreover, treatment interventions have mostly employed varying intensities of aerobic exercise (Colledge et al., 2018a,b). While Menglu et al. (2021a,b) has implemented multiple repetitions of short-interval high-intensity interval training to observe a reduction in drug craving through Stroop cognitive tasks, evidence regarding the effects of anaerobic exercise on SUD patients remains limited (Colledge et al., 2018a,b).

Regarding emotional improvement, 30 min of exercise or rest has been found to significantly improve mood during SUD treatment (Brellenthin et al., 2021). Exercise can also alleviate negative emotional states experienced during withdrawal (Torok et al., 2023). For example, Tai Chi has a significant impact on the protracted abstinence syndrome (PAS) in female heroin-dependent individuals (Li et al., 2013). Further, previous research has found that aerobic exercise can improve anxiety and reduce the risk of depression in SUD patients (Elibero et al., 2011). In individuals suffering from alcohol use disorders, physical activity promoted psychological well-being by improving their emotions, enhancing self-efficacy, and reducing the risk of depression (Sari et al., 2019). The inclusion of physical exercise in the treatment of opioid use disorder significantly reduces depression and anxiety (Durmus et al., 2020). Zhu et al. (2018) suggested that long-term Tai Chi exercise has a positive impact on depression and overall health in amphetamine-type stimulant use disorder (ATS) patients.

The brain disease model of addiction suggests that the risk of SUD recurrence is related to cognitive impairments (Koob and Volkow, 2016). Prolonged drug abuse results in significantly reduced activation of the prefrontal and dorsolateral prefrontal cortex neurons, which are associated with cognitive functions related to drug-seeking behaviors. Long-term withdrawal symptoms and post-withdrawal negative emotions such as anxiety and depression are also factors contributing to relapse (Perry and Carroll, 2008; Goldstein and Volkow, 2011). Therefore, improving cognitive function and alleviating emotional distress are considered effective strategies for SUD patients. Physical exercise has been shown to affect brain cortical plasticity and enhance cognitive function (Gomez-Pinilla and Hillman, 2013). It has also been found to reduce attentional bias in SUD patients, improving cognitive abilities (Zhao et al., 2021). Cognitive functions primarily encompass seven domains: visuospatial and executive functions, naming, memory, attention, language, abstract thinking, and orientation (Tiffin-Richards et al., 2014). Colledge et al. (2018a,b) and Janse Van Rensburg et al. (2009) suggested that physical exercise can effectively improve behavioral performance related to such cognitive functions (e.g., inhibitory control, attentional bias, and working memory), as well as enhance the activity of relevant brain cortical regions, including the prefrontal and dorsolateral prefrontal cortex. Three months of Tai Chi training was found to enhance cognitive abilities, as well as maintain working memory and cognitive flexibility in SUD patients (Shen et al., 2021). Meng and Xue (2021) found that 24 weeks of moderate-intensity aerobic physical exercise had a positive impact on perceptual-attentional function. Zhu et al. (2022) conducted a study on methamphetamine-dependent patients (MA) and found that 12 weeks of moderate-intensity aerobic exercise, in addition to standard withdrawal treatment, significantly promoted the recovery of the blood–brain barrier, brain neurons, and inhibitory control.

While prior research has indicated that physical exercise improves the emotions and cognitive function of SUD patients, different intervention schemes (e.g., exercise type, duration, frequency, and intensity) were employed without identifying the optimal physical exercise intervention. Therefore, this study primarily used a meta-analytical approach to determine the best exercise regimen for enhancing the emotional and cognitive levels of SUD patients. This study contributes to intervention research on SUD. By examining the effects of exercise intervention on the emotions and cognition of individuals with SUD, we can enhance our comprehension of the psychological mechanisms underlying substance use disorders. This, in turn, enables us to offer theoretical support and practical guidance for implementing more effective intervention measures.

## 2 Method

This meta-analysis was carried out following the PRISMA (Preferred Reporting Items for Systematic Reviews and Meta-Analyses) guidelines (Page et al., 2021). The inclusion criteria were designed based on the PICOS (population, intervention, comparison, outcomes, study design) model (Amir-Behghadami and Janati, 2020).

### 2.1 Inclusion and exclusion criteria

Included studies had the following characteristics: (1) Population: studies with a study population comprising SUD patients; (2) Study design: randomized controlled trials (RCTs) evaluating the impact of physical exercise interventions on SUD patients; and (3) Intervention: studies that involved an experimental group utilizing physical exercise interventions, while the control group employed conventional treatment methods or took no action, including irregular physical exercise; (4) Outcome: The primary outcome indicators included scales of emotion: Self-rating Anxiety Scale (SAS), Hamilton Anxiety Rating Scale (HAMA), Beck Anxiety Inventory (BAI), Hospital Anxiety and Depression Scale (HAD), Self-rating Depression Scale (SDS), Beck Depression Inventory (BDI), Hamilton Mood Scale for Depression (HMSD); as well as scales of cognition: Stroop task to evaluate inhibition control, reaction time (RT), and attention bias.

Excluded studies were the following: (1) studies that had non-SUD populations; (2) studies where the changes in indicators for the experimental and control groups pre- and post-physical exercise intervention are unclear; (3) gray literature that remains unpublished or unfinished experiments; and (4) conference abstracts and review studies with incomplete full-text information.

### 2.2 Search strategy

A computer-based search was conducted in five databases: PubMed, ScienceDirect, EBSCO, Web Of Science, and The Cochrane Library. The search spanned from the inception of the databases up to May 2023, without restrictions on publication dates or languages. The following terms were used in the database search: Exercises, Physical Activity, Physical Exercise, Acute Exercise, Isometric Exercise, Aerobic Exercise, Exercise Training, Substance Use Disorder, Substance Related Disorder, Drug Use Disorder, Substance Abuse, Substance Dependence, Substance Addiction, Chemical Dependence, Drug Dependence, Drug Addiction, Prescription Drug Abuse, Substance Use, Drug Abuse, Drug Habituation, Substance Use Disorder, Depression, Anxiety, Mental Health, Mental Problem, and more.

### 2.3 Literature screening and data extraction

Two independent reviewers screened the relevant literature. Based on the inclusion and exclusion criteria, the initial assessment began with a review of the titles and abstracts to eliminate irrelevant studies. Afterward, the remaining studies underwent full-text reading. Both reviewers independently assessed and cross-checked the information. In cases of disagreement, a third party was consulted to assist in reaching a consensus. The review was also extended to all included studies and relevant reference lists to identify potential additional related research.

Data extraction encompassed the following:

- Background information of the included studies (authors, year, country, etc.).
- Baseline data for the study population (sample size, age, gender, etc. for each group).
- Intervention measures (intervention type, duration, frequency, etc.).
- Key factors for assessing bias risk.
- Measurement of outcomes and relevant outcome indicators.

### 2.4 Bias risk assessment for included studies

Two independent reviewers assessed the quality assessment of the included literature using the Cochrane risk of bias tool for RCTs. The assessment focused primarily on the following seven indicators: random sequence generation, blinding of participants and personnel, allocation concealment, blinding of outcome assessors, selective reporting of study results, completeness of outcome data, and other sources of bias.

### 2.5 Statistical analysis

Meta-analysis was conducted using Review Manager 5.4 and Stata 16.0 software. The studies employed a random-effects model, with the standardized mean difference (SMD) as the effect measure. Each effect size was accompanied by its point estimate and a 95% confidence interval (CI). Heterogeneity among the included studies was assessed using the I^2^ statistic, categorized as follows: *I*^2^ = 0–40% (low heterogeneity), *I*^2^ = 30–60% (moderate heterogeneity), *I*^2^ = 50–90% (substantial heterogeneity), and *I*^2^ = 75–100% (considerable heterogeneity). A *p*-value less than 0.05 indicated statistical significance.

To mitigate the impact of heterogeneity on the reporting of results, subgroup analyses were conducted based on the type, duration, frequency, and time of exercise intervention. Exercise intervention contents were categorized as follows: aerobic exercise and Tai Chi (TC) exercise. According to ACSM’s guidelines for exercise testing and prescription (American College of Sports Medicine, 2013), long-term benefits of exercise usually take more than 12 weeks before they occur. Therefore, the intervention cycle was categorized as ≥12 weeks and<12 weeks. And an aerobic activity to 60 min of moderate activity was suggested for more health benefits (Department of Health and Children, Health Service Executive, 2009). Thus, each intervention duration was categorized as ≥60 min and<60 min. As for the threshold of intervention frequency, several different frequencies were suggested by previous papers. For example, individuals are suggested to take moderate-intensity exercise for a minimum of 30 min on 5 days each week or vigorous-intensity exercise for a minimum of 20 min on 3 days each week (Haskell et al., 2007). Considering the inconsistency of the recommended threshold and the intervention frequencies involved in the trails included in this meta-analysis, a median of 40 times (with a total cycle of 12 weeks as a reference, i.e., 3–4 times per week) is considered the threshold. Therefore, the intervention frequency was categorized as ≥40 times and<40 times.

## 3 Results

### 3.1 Literature retrieval

**Figure 1:**
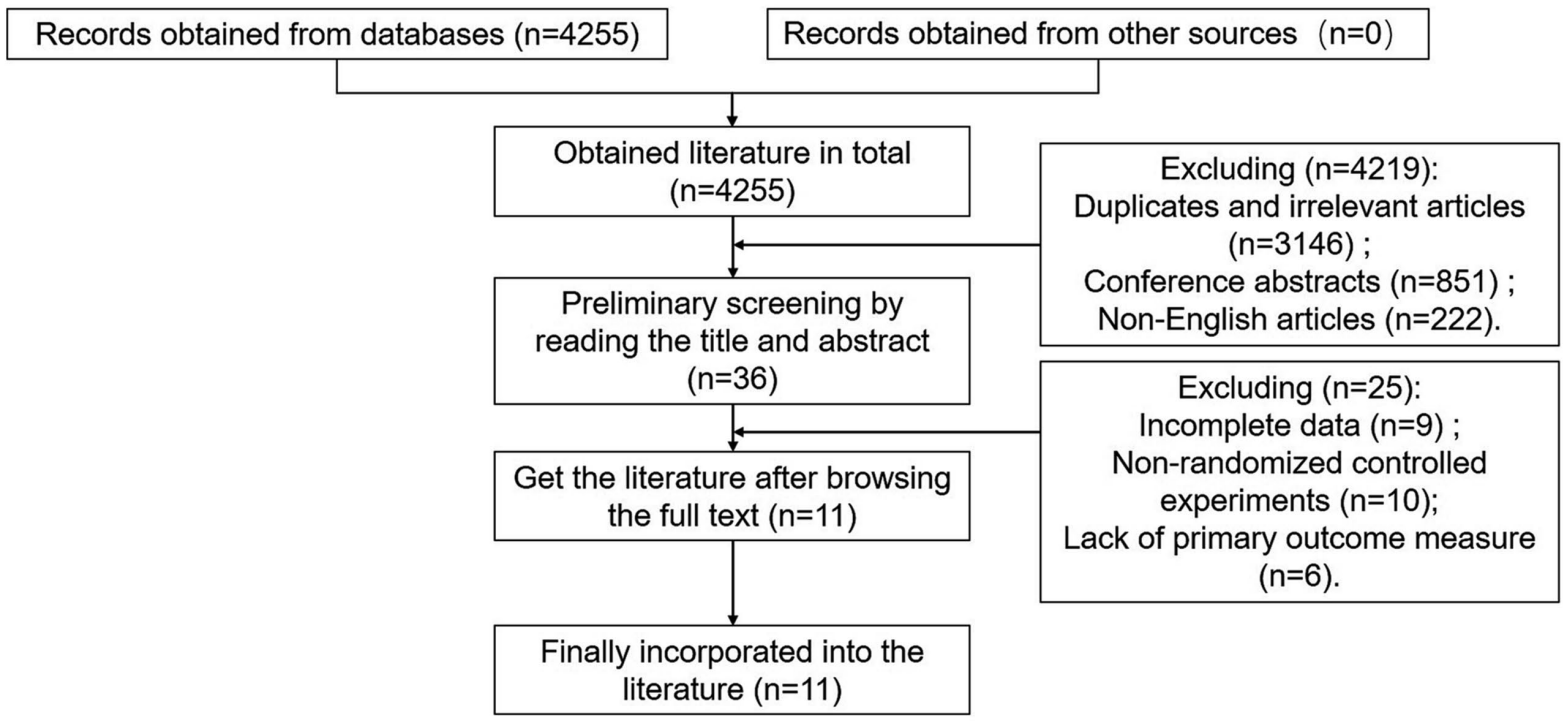
Literature screening process.

A total of 4,255 relevant records were retrieved, of which 4,219 were excluded based on their titles and abstracts during the initial screening. These exclusions comprised duplicates and irrelevant articles (*n* = 3,146), conference abstracts (*n* = 851), and non-English articles (*n* = 222). Further screening by reading the full texts of the remaining 36 literatures led to the exclusion of 25 literatures for reasons such as incomplete data (*n* = 9), non-randomized controlled experiments (*n* = 10), or lack of primary outcome measures (*n* = 6). Finally, 11 literatures were included in the meta-analysis. The selection process and results are summarized in Figure 1.

### 3.2 Basic characteristics of included studies and biased risk assessment

**Figure 2:**
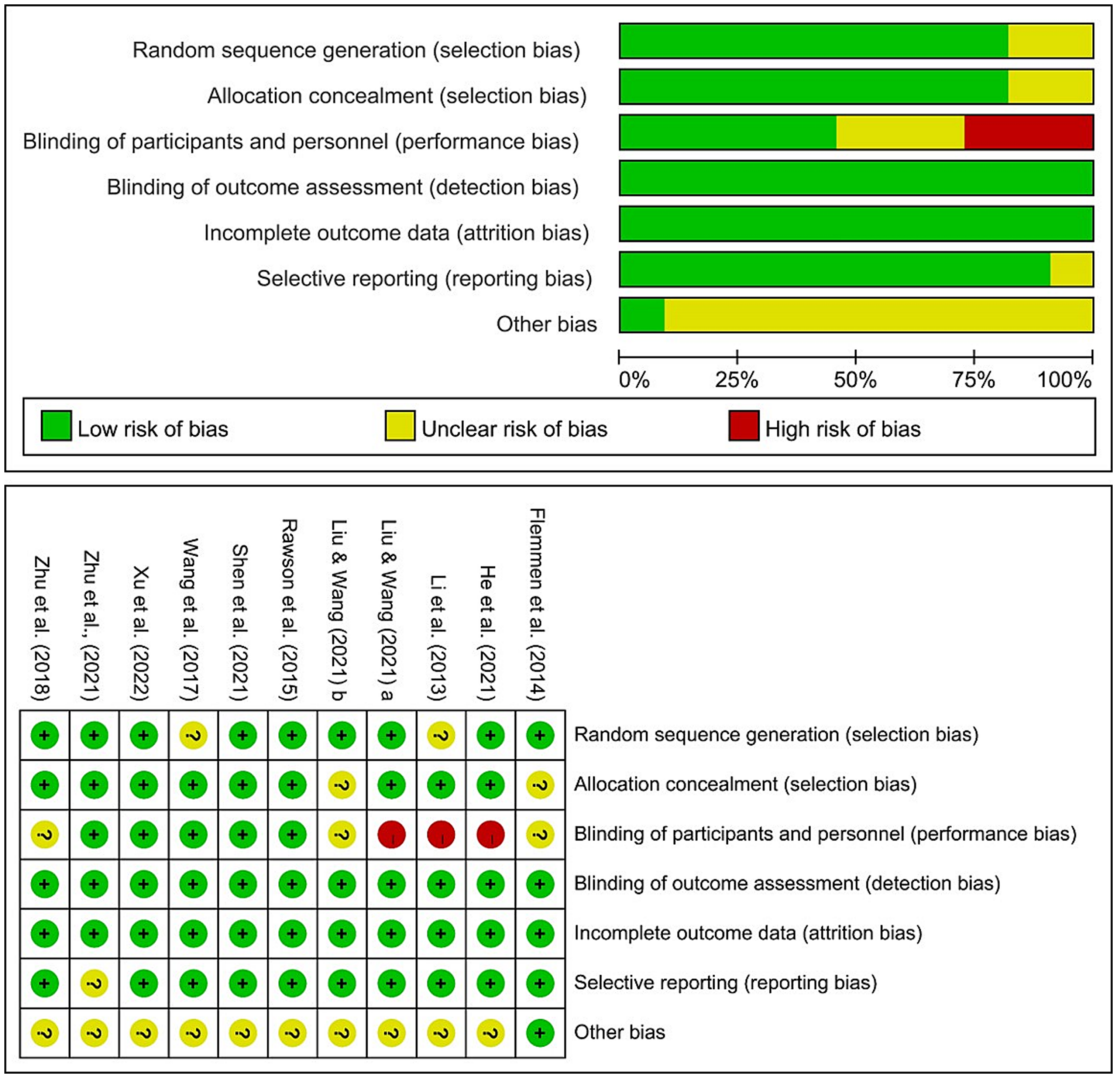
Risk of bias of the included studies.

The basic characteristics of the literature included in the meta-analysis are shown in Table 1, and the results of the bias risk assessment are shown in Figure 2.

**Table 1:** Characteristics of the included studies.

First author (year)	Country	Mean age	Sex	Drug use	Intervention content	Intervention cycle	Intervention frequency	Length of each intervention (session)	Outcome	Experimental group/n	Control group/n
Li et al. (2013)	China	30.7	Female	Heroin addiction	TC	24 weeks	3 times per weeks	80 min	HMSD	17	16
Flemmen et al. (2014)	Norway	32	Both	NA	High-intensity aerobic exercise	8 weeks	3 times per weeks	28 min	HAD	9	7
Rawson et al. (2015)	USA	31.7	Both	Methamphetamine	Aerobic exercise	8 weeks	3 times per weeks	55 min	BDI BAI	69	66
Wang et al. (2017)	China	33.48	Both	Methamphetamine	Moderate-intensity aerobic exercise	12 weeks	3 times per weeks	30 min	RT	25	25
Zhu et al. (2018)	China	35.75	Female	Amphetamine-Type Stimulants	TC	12–24 weeks	3–5 times per weeks	60 min	SDS	42	38
He et al. (2021)	China	42.34	Male	Methamphetamine	TC	4 weeks	3 times per weeks	45 min	RT VAS	16	16
Liu and Wang (2021a,b)	China	31.095	Male	Methamphetamine	Aerobics program	8 weeks	5 times per weeks	60 min	RT	142	146
Liu and Wang (2021a,b)	China	28.40	Male	Methamphetamine	Moderate-intensity aerobic exercise	8 weeks	5 times per weeks	60 min	RT SAS SDS VAS	23	23
Shen et al. (2021)	China	39.34	Female	Methamphetamine	TC	12 weeks	3 times per weeks	40 min	RT	35	37
Zhu et al. (2021)	China	37.78	Male	Methamphetamine	Group-based aerobic exercise	12 weeks	5 times per weeks	30 min	RT HAMA BDI VAS	42	41
Xu et al. (2022)	China	30.4	NA	Methamphetamine	Moderate-intensity aerobic exercise	12 weeks	5 times per weeks	60 min	SAS SDS	30	30

TC, Tai chi; SAS, Self-rated Anxiety Scale; HAMA, Hamilton Anxiety Scale; BAI, Beck Anxiety Inventory; HAD, Hospital Anxiety and Depression Scale; SDS, Self-rating Depression Scale; BDI, Beck Depression Inventory; HMSD, Hamilton Depression Scale; RT, reaction time.

### 3.3 Meta-analysis

#### 3.3.1 Impact of physical exercise on anxiety in SUD patients

The meta-analysis of the impact of physical exercise on anxiety in SUD patients included five studies (*n* = 340). Heterogeneity testing of these five studies showed an SMD of −0.726 [−1.349, −0.103] (Table 2), with a *p*-value of less than 0.05, suggesting that compared to the control group, physical exercise can alleviate anxiety in SUD patients.

**Table 2:** Meta-analysis of the impact of physical exercise on anxiety in SUD patients.

Study (years)	Effect	[95% Conf. Interval]%	
Flemmen et al. (2014)	−0.788	−1.818	0.242
Rawson et al. (2015)	−0.451	−0.793	−0.110
Liu and Wang (2021a,b)	0.158	−0.421	0.737
Zhu et al. (2021)	−0.651	−1.093	−0.209
Xu et al. (2022)	−1.992	−2.615	−1.370
Overall, DL	−0.726	−1.349	−0.103

**Figure 3:**
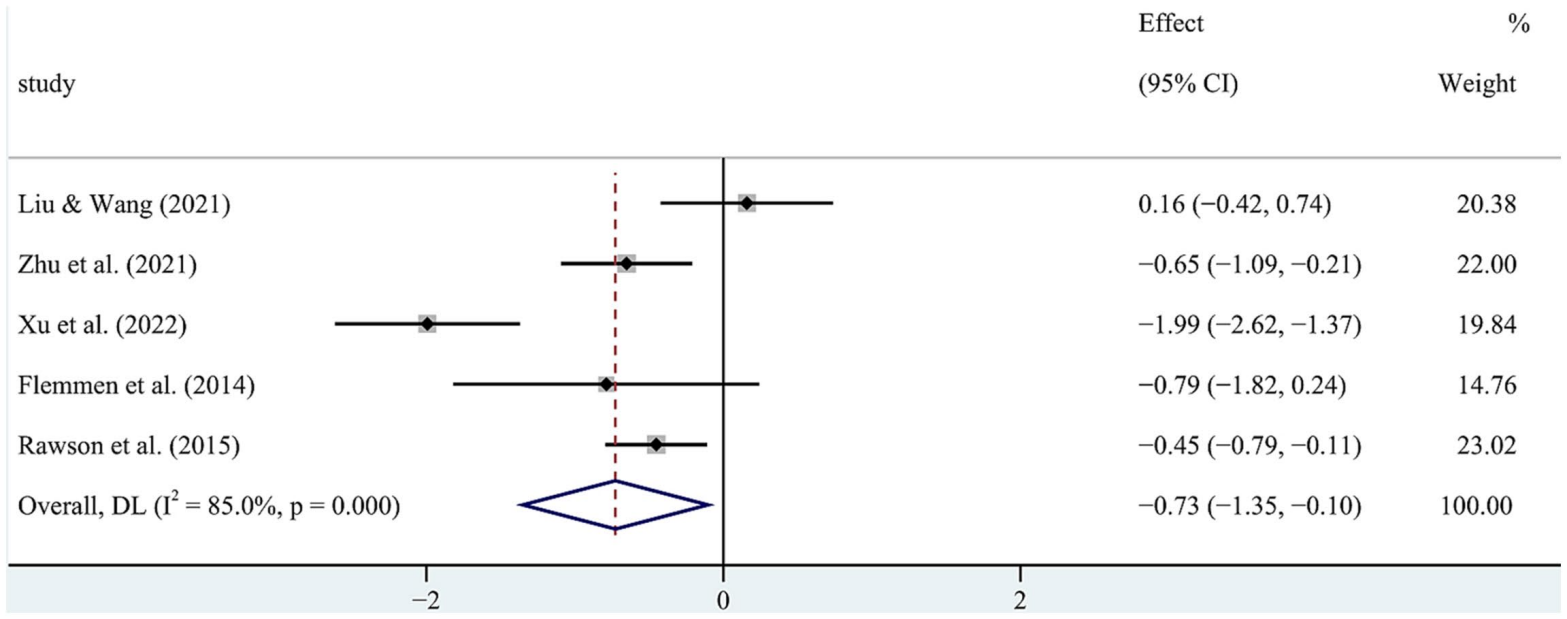
Forest map of the impact of physical exercise on anxiety in SUD patients.

**Figure 4:**
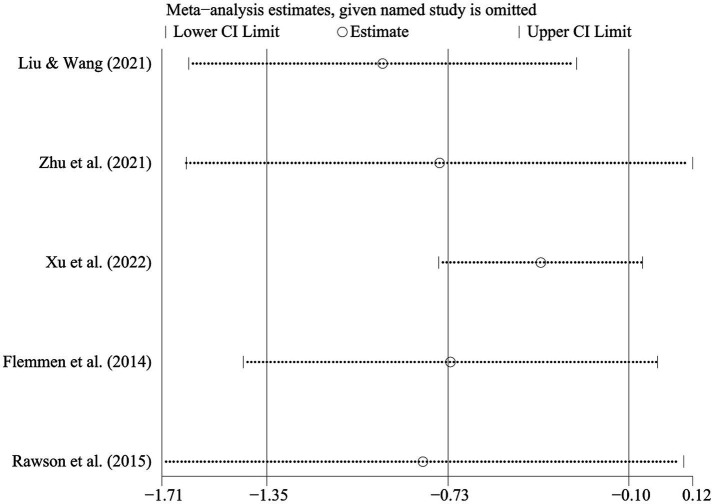
Sensitivity analysis of the impact of physical exercise on anxiety in SUD patients.

The I^2^ statistic was 85.0% (Figure 3), indicating significant heterogeneity. Therefore, a random-effects model was selected for the meta-analysis to explore the sources of heterogeneity. Sensitivity analysis of the five studies (Figure 4) demonstrated overall consistency in the included studies. The results of sensitivity analysis indicated the meta-analysis estimate from other included studies while that given name study is omitted in each line.

In order to further explore sources of heterogeneity, subgroup analyses were conducted regarding intervention content, cycle, and frequency, as well as the length of each exercise session (Table 3). Among the included studies, only aerobic exercise intervention demonstrated statistical significance. Regarding intervention cycle (≥12 weeks; SMD = −1.303 [−2.617, 0.011]), intervention frequency (≥40 sessions; SMD = −0.821 [−1.930, 0.288]), and the length of each intervention session (≥60 min per session; SMD = −0.914 [−3.022, 1.194]), optimal effects were observed. This further confirms the beneficial regulatory effect of physical exercise on anxiety symptoms in SUD patients.

**Table 3:** Subgroup analysis of the impact of physical exercise on anxiety in SUD patients.

Group	Study	Heterogeneity		Results of meta-analysis		
		*I* ^2^	*P*	SMD, 95%CI	*Z*	*P*
Total	5	85%	0.000	−0.726 (−1.349, −0.103)	−2.285	0.022
Intervention content and study (years)						
Aerobic exercise	5	85%	0.000	−0.726 (−1.349, −0.103)	−2.285	0.022
TC	–	–	–	–	–	–
Intervention duration, week						
≥12	2	91.6%	0.001	−1.303 (−2.617, 0.011)	−1.944	0.052
<12	3	49.7%	0.137	−0.300 (−0.777, 0.177)	−1.234	0.217
Guided intervention frequency, times per week						
≥40	3	92.0%	0.000	−0.821 (−1.930, 0.288)	−1.450	0.147
<40	2	0.0%	0.544	−0.485 (−0.809, −0.160)	−2.929	0.003
Length of each intervention session, min						
≥60	2	95.9%	0.000	−0.914 (−3.022, 1.194)	−0.850	0.395
<60	3	0.0%	0.697	−0.543 (−0.805, −0.282)	−4.070	0.000

#### 3.3.2 Impact of physical exercise on depression in SUD patients

**Figure 5:**
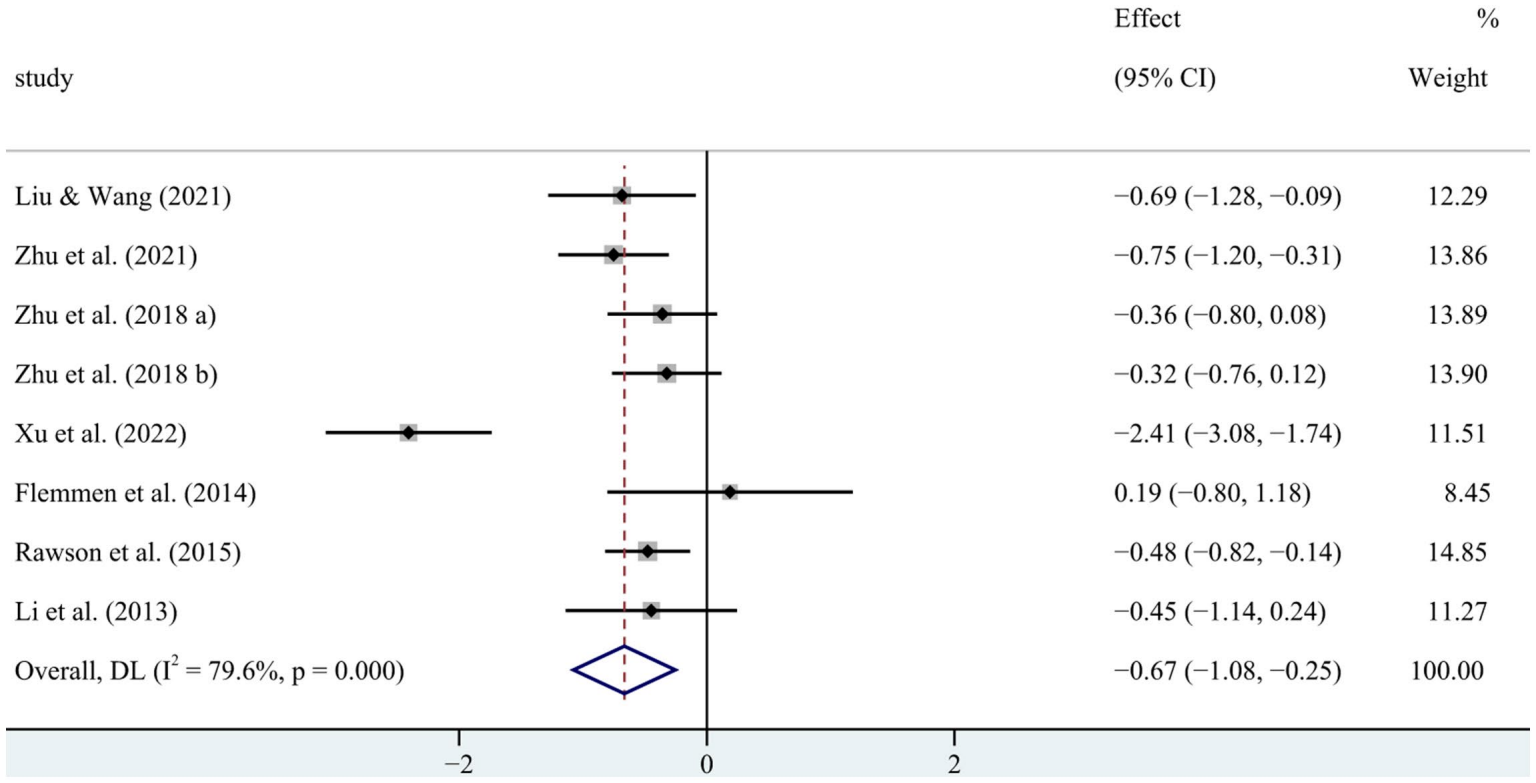
Forest map of the impact of physical exercise on depression in SUD patients.

The meta-analysis on the impact of physical exercise on depression among SUD patients (Table 4) included eight studies (*n* = 533). Heterogeneity testing revealed that SMD = −0.666 [−1.077, −0.255], *p* < 0.05, and *I*^2^ = 79.6%, signifying substantial heterogeneity. Consequently, a random-effects model was chosen for the meta-analysis to explore the sources of heterogeneity. The sensitivity analysis of the seven studies (Figure 5) indicated consistent overall findings when each study was sequentially removed from the analysis.

**Table 4:** Meta-analysis of the impact of physical exercise on depression in SUD patients.

Study (years)	Effect	[95% Conf. Interval] %	
Li et al. (2013)	−0.449	−1.140	0.243
Flemmen et al. (2014)	0.187	−0.803	1.178
Rawson et al. (2015)	−0.478	−0.820	−0.135
Zhu et al. (2018)	−0.359	−0.802	0.083
Zhu et al. (2018)	−0.323	−0.765	0.119
Liu and Wang (2021a,b)	−0.685	−1.281	−0.090
Zhu et al. (2021)	−0.753	−1.199	−0.308
Xu et al. (2022)	−2.408	−3.077	−1.738
Overall, DL	−0.666	−1.077	−0.255

**Figure 6:**
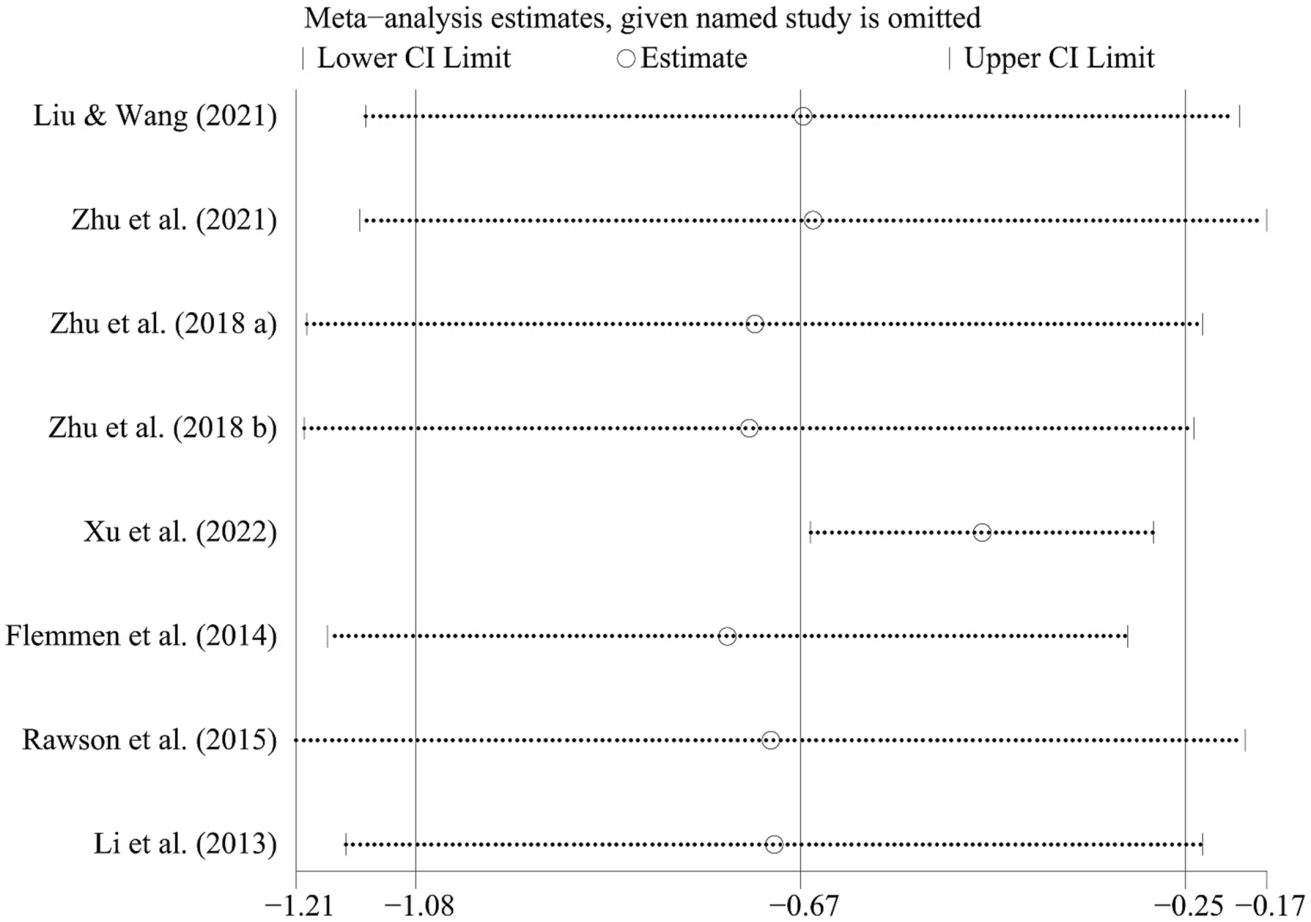
Sensitivity analysis of the impact of physical exercise on depression in SUD patients.

To further investigate the sources of heterogeneity in the impact of physical exercise on depression among SUD patients (Figure 6), subgroup analyses were conducted based on intervention content, cycle, and frequency, as well as the length of each exercise session. The findings revealed the following (Table 5): Optimal exercise modalities for SUD patients’ depression were aerobic exercise (SMD = −0.850 [−1.515, −0.185]), intervention cycle of ≥12 weeks (SMD = −0.832 [−1.473, −0.191]), intervention frequency of ≥40 sessions (SMD = −0.804 [−1.338, −0.269]), and an exercise duration of ≥60 min per session (SMD = −0.822 [−1.503, −0.142]). These results indicate a significant improvement in depression among SUD patients through physical exercise.

**Table 5:** Subgroup analysis of the impact of physical exercise on depression in SUD patients.

Group	Study	Heterogeneity		Results of meta-analysis		
		*I* ^2^	*P*	SMD, 95%CI	*Z*	*P*
Total	8	79.6%	0.000	−0.666 (−1.077, −0.255)	−3.173	0.002
Intervention content and study (years)						
Aerobic exercise	5	86.4%	0.000	−0.850 (−1.515, −0.185)	−2.505	0.012
TC	3	0.0%	0.956	−0.666 (−1.077, −0.255)	−2.473	0.013
Intervention duration, week						
≥12	5	86.9%	0.000	−0.832 (−1.473, −0.191)	−2.545	0.011
<12	3	9.0%	0.333	−0.466 (−0.779, −0.152)	−2.909	0.004
Guided intervention frequency, times per week						
≥40	6	83.7%	0.000	−0.804 (−1.338, −0.269)	−2.948	0.003
<40	2	35.4%	0.213	−0.314 (−0.875, 0.247)	−1.096	0.273
Length of each intervention session, min						
≥60	5	86.9%	0.000	−0.822 (−1.503, −0.142)	−2.369	0.018
<60	3	34.8%	0.215	−0.507 (−0.865, −0.148)	−2.773	0.006

#### 3.3.3 Impact of physical exercise on cognition in SUD patients

**Figure 7:**
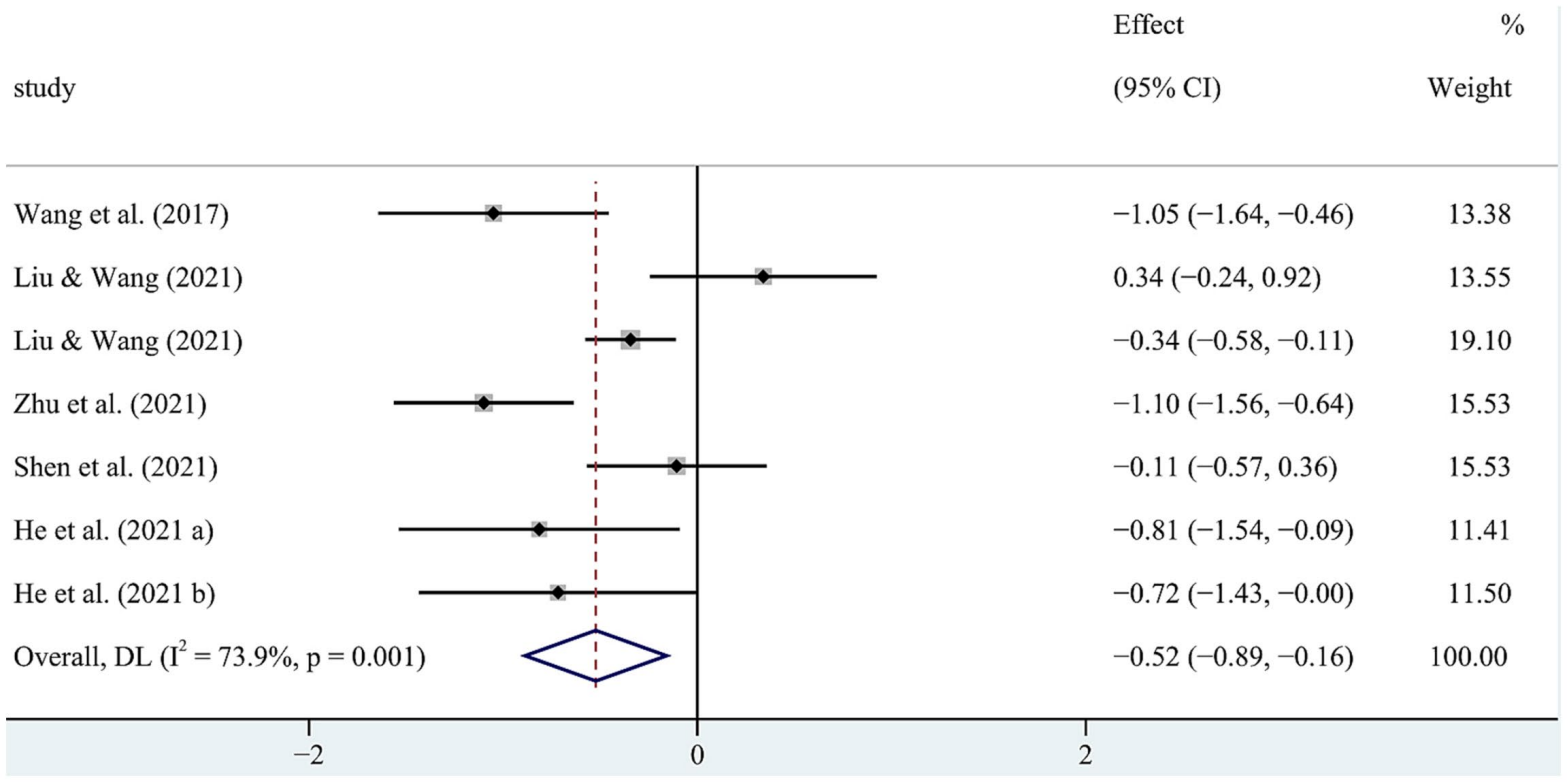
Forest map of the cognitive impact of physical exercise on SUD patients.

**Figure 8:**
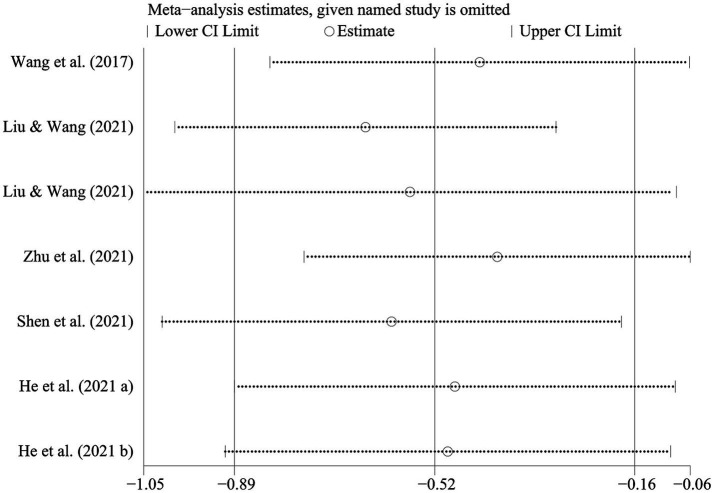
Sensitivity analysis of the cognitive impact of physical exercise on SUD patients.

The meta-analysis on the cognitive impact of physical exercise on SUD patients (Table 6) included seven studies (*n* = 603). Heterogeneity analysis indicated that SMD = −0.523 [−0.887, −0.159], *p* < 0.05, and *I*^2^ = 73.9%, signifying substantial heterogeneity. Consequently, a random-effects model was utilized for the meta-analysis to explore the sources of heterogeneity (Figure 7). Sensitivity analysis conducted on the studies revealed consistent overall findings when each study was sequentially removed from the analysis (Figure 8).

**Table 6:** Meta-analysis of the cognitive impact of physical exercise on SUD patients.

Study (years)	Effect	[95% Conf. Interval] %	
Wang et al. (2017)	−1.050	−1.643	−0.457
He et al. (2021)	−0.814	−1.537	−0.091
He et al. (2021)	−0.718	−1.434	−0.001
Liu and Wang (2021a,b)	0.339	−0.243	0.921
Liu and Wang (2021a,b)	−0.344	−0.577	−0.111
Shen et al. (2021)	−0.107	−0.570	0.355
Zhu et al. (2021)	−1.100	−1.563	−0.638
Overall, DL	−0.523	−0.887	−0.159

To further explore the sources of heterogeneity in the cognitive impact of physical exercise on SUD patients, subgroup analyses were conducted based on intervention content, cycle, and frequency, as well as the length of each exercise session (Table 7). Among the intervention schemes, aerobic exercise (SMD = −0.539 [−1.101, 0.023]), an intervention cycle of ≥12 weeks (SMD = −0.743 [−1.408, 0.077]), and an intervention frequency of ≥40 sessions (SMD = −0.387 [−1.047, 0.273]) demonstrated more significant improvements in cognitive effects among SUD patients. However, an intervention duration of ≥60 min per session (SMD = −0.057 [−0.718, 0.604]) did not show significant effects on cognitive improvement.

**Table 7:** Subgroup analysis of the cognitive impact of physical exercise on SUD patients.

Group	Study	Heterogeneity		Results of meta-analysis		
		*I* ^2^	*P*	SMD, 95%CI	*Z*	*P*
Total	7	73.9%	0.001	−0.523 (−0.887, −0.159)	−2.816	0.005
Intervention content and study (years)						
Aerobic exercise	4	84.5%	0.000	−0.539 (−1.101, 0.023)	−1.881	0.060
TC	3	43.8%	0.956	−0.474 (−0.953, −0.005)	−1.941	0.052
Intervention duration, week						
≥12	3	81.0%	0.005	−0.743 (−1.408, 0.077)	−2.186	0.029
<12	4	62.2%	0.047	−0.345 (−0.776, 0.087)	−1.567	0.117
Guided intervention frequency, times per week						
≥40	3	86.9%	0.000	−0.387 (−1.047, 0.273)	−1.150	0.250
<40	4	57.0%	0.073	−0.636 (−1.102, −0.169)	−2.669	0.008
Length of each intervention session, min						
≥60	2	78.1%	0.033	−0.057 (−0.718, 0.604)	−0.168	0.866
<60	5	62.3%	0.031	−0.746 (−1.165, −0.327)	−3.493	0.000

## 4 Discussion

SUD can lead to severe health problems, exerting negative impacts on the lives of addicts (Kohlerova et al., 2023). These disorders deplete dopamine storage in the brain, triggering withdrawal reactions and adverse effects such as anxiety, depression, and cognitive decline, resulting in severe negative emotional states (Brellenthin and Koltyn, 2016). Physical exercise, emerging as a novel intervention for patients with SUD, has proven effective (Dowla et al., 2022). A previous systematic review from Piché et al. (2023) has suggested that most common physical activity intervention during SUD treatment identified was of moderate intensity, 3 times per week (≈ 1 h) for 13 weeks, leads to a similar conclusion of the present study. Another meta-analysis on the effects of exercise of different intensities on withdrawal symptoms among people with SUD indicated that different intensity exercise reduced depression after the intervention with moderate-intensity exercise producing the best effect, while only light- and moderate-intensity exercise relieved anxiety (Li et al., 2023). Although this meta-analysis discussed the effects of exercise of different intensities, it did not consider the duration, frequency, type, or period of the exercise interventions. Despite limited meta-analyses on the effects of SUD on emotions and cognition, our study, based on a meta-analysis of randomized controlled trials, demonstrates that physical exercise can significantly reduce anxiety and depression in SUD patients and improve cognitive levels, and explore the most effective intervention types, cycle, frequency, and duration in order to provide evidence-based recommendations for the adjunctive treatment of SUD.

Various pieces of evidence indicate a correlation between depression and physical activity, suggesting that exercise intervention can impact the circulating levels of neurotransmitters (serotonin and dopamine) in methamphetamine addicts, thereby reducing anxiety and depression in patients (Arazi et al., 2022; Berger et al., 2023). Moreover, physical exercise diminishes the sensitivity and attentional bias of patients to drug-related cues, signifying that exercise may facilitate cognitive recovery in patients through attentional control (He et al., 2021).

There are primarily 10 types of drugs that activate the brain’s reward system and induce pleasure through material use disorders (Sherer et al., 2023). Previous studies have demonstrated the beneficial effects of aerobic exercise on users of opioids (Jake-Schoffman et al., 2020) and amphetamines (Zhang and Liu, 2022). This meta-analysis primarily includes participants with methamphetamine addiction, reaffirming the positive impact of exercise intervention on the emotions and cognition of such patients.

## 5 Conclusion

Current therapeutic methods for treating SUD include spiritual support (Volenik, 2021), psychotherapy (Ondersma et al., 2009), medication (Hasebe et al., 2004), and exercise intervention. Compared to traditional treatment methods, exercise interventions not only lower the risk of cardiovascular diseases and premature death among SUD patients (Dowla et al., 2022), but they have also been found to result in substantial improvements in patients’ emotional and cognitive levels. This study contributes to the research in the field of substance use disorders, aiming to offer more effective solutions for their treatment. By conducting a thorough and precise assessment of the influence of exercise intervention on individuals with SUD with the approach of meta-analysis, we strive to advance the systematization and integration of the treatment on SUD from the perspective of psychological research.

This meta-analysis aimed to investigate the impact of physical exercise on the emotional and cognitive levels of SUD patients. Eleven RCT articles related to the effects of physical exercise on the emotional and cognitive levels of SUD patients were selected from databases including PubMed, Web Of Science, The Cochrane Library, ScienceDirect, and EBSCO. Current research findings suggest that physical exercise can alleviate anxiety and depression symptoms among SUD patients while enhancing cognitive function. Hence, exercise interventions can serve as a supplementary treatment approach in the therapy of SUD patients. Overall, physical exercise reduces anxiety (SMD = −0.726 [−1.349, −0.103], *p* < 0.05) and depression (SMD = −0.666 [−1.077, −0.255], *p* < 0.05), and improves cognitive levels (SMD = −0.523 [−0.887, −0.159], *p* < 0.05) among SUD patients. Subgroup analysis revealed that aerobic exercise sustained for over 12 weeks, conducted at a frequency of more than 40 sessions, each lasting over 60 min, significantly enhances emotional and cognitive improvements in SUD patients, indicating the importance of long-term exercise interventions for improved therapeutic outcomes.

This study has certain limitations. First, only 11 studies with a total of 895 participants met the inclusion criteria of this meta-analysis. And the majority of the studies were conducted in China, introducing potential regional limitations to the generalizability of the research results. To address this, future research endeavors should involve more comprehensive and high-quality randomized controlled trials conducted in diverse cultural backgrounds. This will facilitate discussions on the applicability of different intervention plans in various cultural and social contexts. We are committed to staying abreast of the latest developments in related research, providing a reliable foundation for updating research in this field. Second, we could not explore the grouping of different types of SUD because of the limited sample size. In upcoming research, we suggest to categorize SUD based on their types (such as opioids, amphetamines, cocaine, and alcohol) in order to explore the distinct impact of aerobic exercise on these different types of SUD, allowing for the formulation of targeted intervention plans. Besides, in the present meta-analysis, we mainly focused on outcomes such as reaction time and attention bias to capture the cognitive functions of SUD patients. However, impairments in memory are still a significant consequence of SUD (Yan et al., 2014). Future studies may consider conducting subgroup analysis to assess intervention effects on patients’ working and episodic memory. Despite limitations such as the exclusion of gray literature and relatively small sample sizes, the positive impact of physical exercise on the emotional and cognitive levels of SUD patients is evident. In conclusion, exercise plays a significant role in assisting SUD treatments. However, more evidence and research are needed to further confirm and refine these findings for better treatment planning. Future research should thus focus on strengthening exercise intervention strategies to develop more scientifically grounded treatment approaches.

## Data availability statement

The original contributions presented in the study are included in the article/supplementary material, further inquiries can be directed to the corresponding authors.

## Author contributions

YaZ: Conceptualization, Formal analysis, Investigation, Writing – original draft. YiZ: Data curation, Software, Writing – original draft. XC: Formal analysis, Methodology, Writing – original draft. SL: Supervision, Validation, Writing – review & editing.
